# Evidence for Community Transmission of Community-Associated but Not Health-Care-Associated Methicillin-Resistant *Staphylococcus Aureus* Strains Linked to Social and Material Deprivation: Spatial Analysis of Cross-sectional Data

**DOI:** 10.1371/journal.pmed.1001944

**Published:** 2016-01-26

**Authors:** Olga Tosas Auguet, Jason R. Betley, Richard A. Stabler, Amita Patel, Avgousta Ioannou, Helene Marbach, Pasco Hearn, Anna Aryee, Simon D. Goldenberg, Jonathan A. Otter, Nergish Desai, Tacim Karadag, Chris Grundy, Michael W. Gaunt, Ben S. Cooper, Jonathan D. Edgeworth, Theodore Kypraios

**Affiliations:** 1 Centre for Clinical Infection and Diagnostics Research, Department of Infectious Diseases, King's College London and Guy's and St Thomas' NHS Foundation Trust, London, United Kingdom; 2 Centre for Tropical Medicine and Global Health, Nuffield Department of Clinical Medicine, University of Oxford, Oxford, United Kingdom; 3 Illumina, Cambridge Limited, Chesterford Research Park, Little Chesterford, Essex, United Kingdom; 4 Department of Pathogen Molecular Biology, Faculty of Infectious and Tropical Diseases, London School of Hygiene and Tropical Medicine, London, United Kingdom; 5 Department of Medical Microbiology, King's Hospital NHS Foundation Trust, London, United Kingdom; 6 Department of Microbiology, University Hospital Lewisham, Lewisham and Greenwich NHS Trust, London, United Kingdom; 7 Department of Social and Environmental Health Research, Faculty of Public Health and Policy, London School of Hygiene and Tropical Medicine, London, United Kingdom; 8 Mahidol Oxford Tropical Medicine Research Unit (MORU), Bangkok, Thailand; 9 School of Mathematical Sciences, University Park, University of Nottingham, Nottingham, United Kingdom; The National Institute for Public Health and the Environment, NETHERLANDS

## Abstract

**Background:**

Identifying and tackling the social determinants of infectious diseases has become a public health priority following the recognition that individuals with lower socioeconomic status are disproportionately affected by infectious diseases. In many parts of the world, epidemiologically and genotypically defined community-associated (CA) methicillin-resistant *Staphylococcus aureus* (MRSA) strains have emerged to become frequent causes of hospital infection. The aim of this study was to use spatial models with adjustment for area-level hospital attendance to determine the transmission niche of genotypically defined CA- and health-care-associated (HA)-MRSA strains across a diverse region of South East London and to explore a potential link between MRSA carriage and markers of social and material deprivation.

**Methods and Findings:**

This study involved spatial analysis of cross-sectional data linked with all MRSA isolates identified by three National Health Service (NHS) microbiology laboratories between 1 November 2011 and 29 February 2012. The cohort of hospital-based NHS microbiology diagnostic services serves 867,254 usual residents in the Lambeth, Southwark, and Lewisham boroughs in South East London, United Kingdom (UK). Isolates were classified as HA- or CA-MRSA based on whole genome sequencing. All MRSA cases identified over 4 mo within the three-borough catchment area (*n* = 471) were mapped to small geographies and linked to area-level aggregated socioeconomic and demographic data. Disease mapping and ecological regression models were used to infer the most likely transmission niches for each MRSA genetic classification and to describe the spatial epidemiology of MRSA in relation to social determinants. Specifically, we aimed to identify demographic and socioeconomic population traits that explain cross-area extra variation in HA- and CA-MRSA relative risks following adjustment for hospital attendance data. We explored the potential for associations with the English Indices of Deprivation 2010 (including the Index of Multiple Deprivation and several deprivation domains and subdomains) and the 2011 England and Wales census demographic and socioeconomic indicators (including numbers of households by deprivation dimension) and indicators of population health. Both CA-and HA-MRSA were associated with household deprivation (CA-MRSA relative risk [RR]: 1.72 [1.03–2.94]; HA-MRSA RR: 1.57 [1.06–2.33]), which was correlated with hospital attendance (Pearson correlation coefficient [PCC] = 0.76). HA-MRSA was also associated with poor health (RR: 1.10 [1.01–1.19]) and residence in communal care homes (RR: 1.24 [1.12–1.37]), whereas CA-MRSA was linked with household overcrowding (RR: 1.58 [1.04–2.41]) and wider barriers, which represent a combined score for household overcrowding, low income, and homelessness (RR: 1.76 [1.16–2.70]). CA-MRSA was also associated with recent immigration to the UK (RR: 1.77 [1.19–2.66]). For the area-level variation in RR for CA-MRSA, 28.67% was attributable to the spatial arrangement of target geographies, compared with only 0.09% for HA-MRSA. An advantage to our study is that it provided a representative sample of usual residents receiving care in the catchment areas. A limitation is that relationships apparent in aggregated data analyses cannot be assumed to operate at the individual level.

**Conclusions:**

There was no evidence of community transmission of HA-MRSA strains, implying that HA-MRSA cases identified in the community originate from the hospital reservoir and are maintained by frequent attendance at health care facilities. In contrast, there was a high risk of CA-MRSA in deprived areas linked with overcrowding, homelessness, low income, and recent immigration to the UK, which was not explainable by health care exposure. Furthermore, areas adjacent to these deprived areas were themselves at greater risk of CA-MRSA, indicating community transmission of CA-MRSA. This ongoing community transmission could lead to CA-MRSA becoming the dominant strain types carried by patients admitted to hospital, particularly if successful hospital-based MRSA infection control programmes are maintained. These results suggest that community infection control programmes targeting transmission of CA-MRSA will be required to control MRSA in both the community and hospital. These epidemiological changes will also have implications for effectiveness of risk-factor-based hospital admission MRSA screening programmes.

## Abstract 2

Community associated MRSA variants, rather than hospital associated ones, are more readily transmitted and this is where control programs should focus to limit both hospital and community infections.

## Editors' Summary

**Background:**

Addressing health inequality requires understanding the social determinants of poor health. Previous studies have suggested a link between deprived living conditions and infections with methicillin-resistant *Staphylococcus aureus* (MRSA), that is, strains of the common bacterium *S*. *aureus* that have acquired antibiotic resistance and are therefore more difficult to treat. MRSA was first identified in the 1960s and for years thought of as a dangerous health-care-associated (HA-) pathogen that infects hospital patients who are predominantly older, sick, or undergoing invasive procedures. In the late 1990s, however, community-associated MRSA (CA-MRSA) emerged as pathogen infecting healthy individuals of all ages and without recent hospital contact. Most CA-MRSA cases are contagious skin infections, and numerous outbreaks have been reported in different communities. The traditional distinction between HA-MRSA and CA-MRSA based on where transmission occurred has become problematic in recent years, because CA-MRSA transmission has also been reported in health care settings. However, as HA- and CA-MRSA strains are genetically distinct, cases can be classified by DNA sequencing regardless of where a patient got infected.

**Why Was This Study Done?:**

With hospitals historically considered the only place of MRSA transmission, prevention efforts remain focused on health care settings. Given the changing patterns of MRSA infections, however, the need to consider HA and CA transmission settings together has been recognized. This study was designed to take a closer look at the relationship between both HA- and CA-MRSA and socioeconomic deprivation, with the ultimate aim to inform prevention efforts. The researchers selected three boroughs in South East London with a highly diverse population of approximately 850,000 residents for whom socioeconomic and demographic data were available at a high level of spatial resolution. They also had data on hospital attendance for the residents and were therefore able to account for this factor in their analysis. The study addressed the following questions: is there a link between socioeconomic deprivation and both HA- and CA-MRSA cases among the residents? What social determinants are associated with HA- and CA-MRSA cases? What are the transmission settings (i.e., community versus health care) for HA- and CA-MRSA?

**What Did the Researchers Do and Find?:**

They analyzed data on all MRSA samples collected over 4 consecutive mo in late 2011 and early 2012 by microbiology laboratories that serve the three boroughs. Of 471 MRSA cases that occurred in residents, 392 could be classified based on genome sequencing. Of these, approximately 72% were HA-MRSA, and 26% were CA-MRSA. Approximately 2% of residents carried both HA- and CA-MRSA. All MRSA cases were mapped to 513 smaller areas (called Lower Layer Super Output Areas, or LSOAs) in the three boroughs for which extensive socioeconomic and demographic data existed. The former included data on income, employment, health, and education, the latter data on number individuals per household, their ages and gender, and length of residence in the UK. MRSA cases were detected in just over half of the LSOAs in the study area. The researchers then used mathematical models to determine the most likely transmission settings for each MRSA genetic classification. They also described the spatial distributions of the two in relation to socioeconomic and demographic determinants. Both CA-and HA-MRSA were associated with household deprivation, which was itself correlated with hospital attendance. HA-MRSA was also associated with poor health and with living in communal care homes, whereas CA-MRSA was linked with household overcrowding and a combination of household overcrowding, low income, and homelessness. CA-MRSA was also associated with recent immigration to the UK. Around 27% of local variation in CA-MRSA could be explained by the spatial arrangement of LSOAs, meaning areas of high risk tended to cluster. No such clustering was observed for HA-MRSA.

**What Do these Findings Mean?:**

The results show that residents in the most deprived areas are at greater risk for MRSA. The absence of spatial clusters of HA-MRSA suggests that transmission of genetically determined HA-MRSA occurs in hospitals, with little or no transmission in the community. The most important risk factor for acquiring HA-MRSA is therefore likely to be hospital attendance as a result of deprivation. In contrast, genetically determined CA-MRSA both affects deprived areas disproportionately, and—as the clusters imply—spreads from such areas in the community. This suggests that living in deprived conditions itself is a risk factor for acquiring CA-MRSA, as is living near deprived neighbors. Some of the CA-MRSA cases are also likely imported by recent immigrants. Whereas transmission of CA-MRSA in health care settings has been reported in a number of other studies, data from this study cannot answer whether or to what extent this is the case here. However, because of ongoing transmission in the community, and because deprived residents are both more likely to have CA-MRSA and to attend a hospital, importation of CA-MRSA strains into hospitals is an obvious concern. While the researchers intentionally located the study in an area with a very diverse population, it is not clear how generalizable the findings are to other communities, either in the UK or in other countries. Nonetheless, the results justify special focus on deprived populations in the control of MRSA and are useful for the design of specific strategies for HA-MRSA and CA-MRSA.

**Additional Information:**

Please access these Web sites via the online version of this summary at http://dx.doi.org/10.1371/journal.pmed.1001944.

- Online information on MRSA from the UK National Health Service: http://www.nhs.uk/conditions/MRSA/Pages/Introduction.aspx
- MRSA webpage from the US Centers of Disease Control and Prevention: http://www.cdc.gov/mrsa/
- MRSA page from the San Francisco Department of Public Health: http://www.sfcdcp.org/mrsa.html
- MedlinePlus provides links to information about MRSA, including sources in languages other than English: https://www.nlm.nih.gov/medlineplus/mrsa.html

## Introduction

In recent years, systematic health inequalities and the uneven distribution of adverse health outcomes have been found to affect a wide array of infectious diseases, not just chronic diseases or signature infections of social determinants such as tuberculosis or human immunodeficiency virus (HIV) [1–3]. In 2004, a study in the UK showed that the incidence of postoperative infection with methicillin-resistant *Staphylococcus aureus* (MRSA) was 7-fold higher in patients whose residential postcode was located in the most deprived areas [4]. Following the recognition that individuals with lower socioeconomic status are disproportionately affected by infections in every European Union member state [1], addressing the social determinants of infectious diseases has become a public health priority in recent years [5,6].

The epidemiology of MRSA is complex, particularly given the coexistence of two genetically and epidemiologically distinct classifications. Until the emergence of community-associated MRSA (CA-MRSA) in the late 1990s [7,8], infection was predominantly due to health-care-associated (HA) strains associated with advanced age, comorbidities, surgical procedures, or indwelling medical devices [9–12]. CA-MRSA later emerged as a cause of infection in the community in previously healthy individuals of all ages, with no history of hospital contact and none of the risk profiles that are typical of health care exposure [7,8]. Recently, however, CA-MRSA strains have emerged as a cause of health-care-associated infection in some parts of the world [13], challenging definitions of CA-MRSA based on clinical epidemiology and where disease manifests [14–16] in favour of genotype-based definitions [17–19]. Nonetheless, CA-MRSA strains retain a number of important characteristics, notably the association with infection in previously healthy individuals in the community [7,8,12,16,20].

Health care settings are regarded as the epicentre for MRSA transmission in Europe [21,22]. Consequently, to date the predominant focus in the UK has been HA-MRSA and minimising the threat to patients through rigorous infection control practice and universal admission screening [10,23–25]. However, it is increasingly recognised that this approach is at odds with the shifting epidemiology of MRSA, which makes it necessary to consider both HA- and CA-MRSA transmission dynamics together [26]. Whilst application of advanced spatial statistical methods may be useful to characterise transmission dynamics of MRSA [21,27,28], application of these models to describe the epidemiology in relation to social determinants and health care exposure is lacking.

The objectives of this study are 2-fold: first, to investigate the relationship between socioeconomic deprivation and both CA- and HA-MRSA in South East London and to identify whether social determinants are associated with carriage of either MRSA classification and, second, to identify the main transmission niches for HA- and CA-MRSA (i.e., community versus health care setting) and whether niches are distinct or shared. For the first time, we fit spatial models and account for hospital attendance in each geography to establish the relative contribution of spatial effects and account for any potential confounding.

## Methods

### Study Population

**Fig 1:**
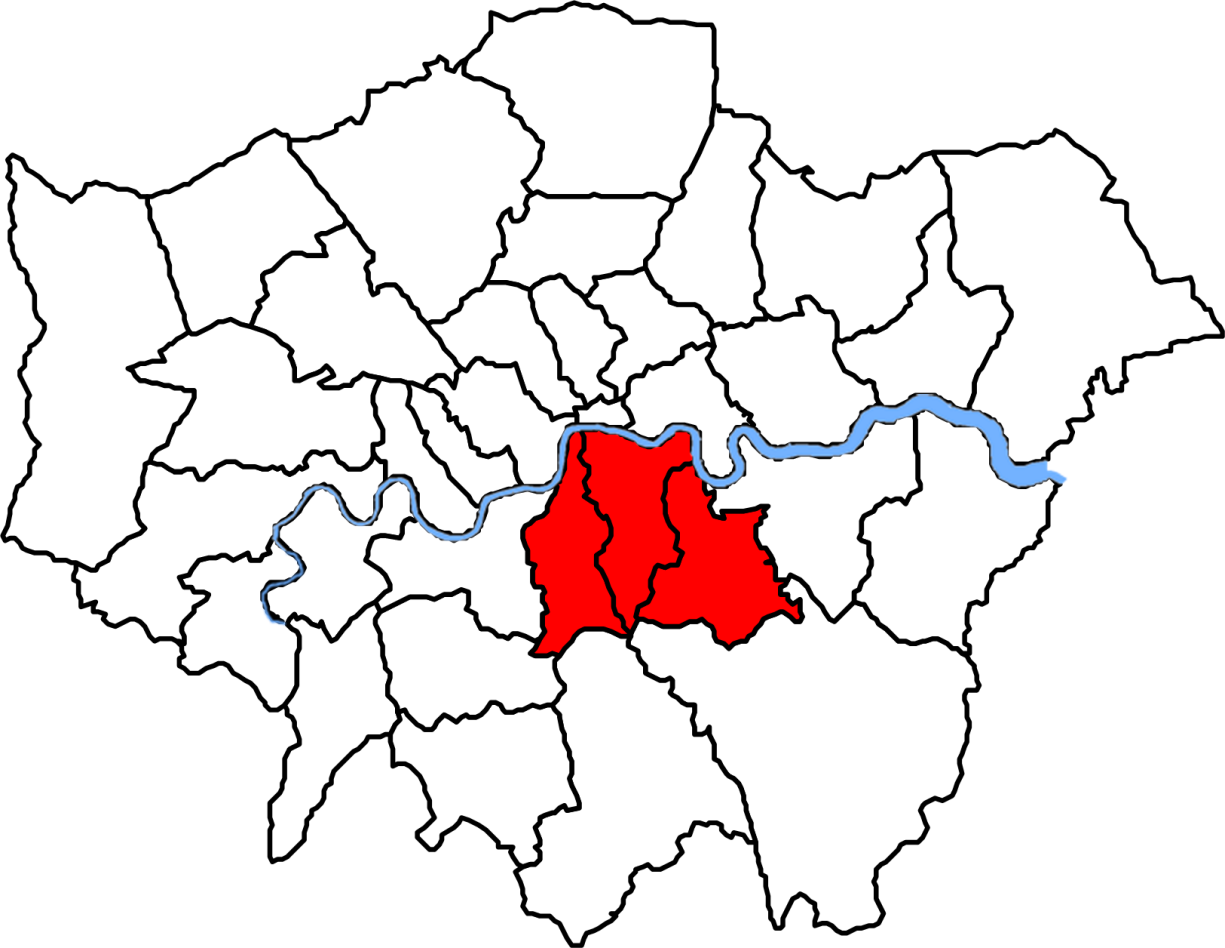
Map of London boroughs showing catchment areas for the hospital cohort. Shown in red from left to right, catchment areas for the hospital cohort were south of the river Thames and included the Southwark, Lambeth, and Lewisham boroughs.

A description of the study population has been reported previously [29]. Briefly, over a 4-mo period (from 1 November 2011 to 29 February 2012), we collected all MRSA isolates identified by a hospital cohort that serves a population of 867,254 usual residents [30] and provides microbiology diagnostic services to all inpatients, outpatients, and community patients in the Lambeth, Southwark, and Lewisham boroughs in South East London (Fig 1). Participant centres included four acute tertiary hospitals in two NHS Trusts (Guy’s and St Thomas’ NHS Foundation Trust [GSTT]; King’s College NHS Foundation Trust) and one acute district general hospital (University Hospital Lewisham; Lewisham and Greenwich NHS Trust).

### Laboratory Methods

MRSA isolates were submitted to the Centre for Clinical Infection and Diagnostics Research (CIDR) at GSTT and were included in the study if confirmed as MRSA by culture on chromogenic agar (Oxoid Brilliance) and rapid latex agglutination test (Staphaurex, Remel) [29]. Relevant clinical, geographic, and demographic anonymised patient-level details were submitted with each specimen and recorded in the study database [29]. Whole genome sequencing (WGS) was conducted on the first confirmed MRSA isolate from each individual at each unique health care setting (i.e., whenever an individual was admitted as inpatient to a new hospital or received care in a new outpatient clinic or community service during the study period), implying availability of follow-up genomic information when patients received care at multiple settings. Genomic DNA quantification, DNA sample preparation, and library pooling for paired-end 150-cycle sequencing on the Illumina HiSeq 2500 platform in rapid run mode was conducted as per manufacturer’s instructions and as described previously [29]. Following de novo assembly of contigs [29], draft assemblies were analysed in silico to determine the multilocus sequence type (MLST), staphylococcal cassette chromosome *mec* (SCC*mec*) type and carriage of the Panton-Valentine leukocidin (PVL) using BWA [31] and BLAST [32]. Isolates were classified as HA-MRSA if they were PVL negative and contained SCC*mec* types I, II, or III, and as CA-MRSA if they were PVL positive or contained type IV, V, or nontypeable SCC*mec* [19,29,33]. Exceptions were ST22-IV isolates and ST5-IV isolates, which were classified as HA unless they were PVL positive [19,29,33]. WGS data are available from the European Nucleotide Archive database under accession number PRJEB11177.

### Data Collection

The study included the subset of all individuals who had a confirmed MRSA isolate, but also a residential postcode within the catchment areas (Fig 1). Eligible individuals with MRSA were then classed as CA- or HA-MRSA based on the WGS analysis. Persons with both CA- and HA-MRSA in repeated samplings were classified as having a mixed infection and excluded from analyses of CA- and HA-MRSA but included in the analysis of all MRSA. Boundary data for mapping 2011 small geographies (named Lower Layer Super Output Areas [LSOAs]) in catchment areas were obtained from the Office for National Statistics, UK [34]. An LSOA is a geography for the collection and publication of small area statistics in England and Wales; each LSOA has 1,500 residents and 650 households on average. MRSA cases were then mapped to LSOAs following conversion of the residential postcode into a LSOA using GeoConvert [35]. Publicly available small-area-level aggregated data for each LSOA within the Lambeth, Southwark, and Lewisham catchment areas (*n* = 513) were obtained from the English Indices of Deprivation 2010 [36] and the 2011 England and Wales census [30]. Demographic data (population census and age and gender population structure) were also obtained from the 2011 census. Publicly available data from these sources that were included in the analysis of potential risk factors of MRSA are given in Box 1. The individual patient-level and aggregated LSOA-level metadata analysed in this study are available in S1 and S2 Text, respectively.

**Box 1. Description of Publicly Available Area-Level Data Utilised in Ecological Regression Models to Assess Risk Factors for Health-Care-Associated (HA-) and Community-Associated (CA-) MRSA**

Summary statistics for area-level variables are given in S1 Table.

**The English Indices of Deprivation 2010 [36]**

The **Index of Multiple Deprivation (IMD)** is a wide measure of multiple deprivation for Lower Layer Super Output Area (LSOA) geographies in England. It is conceptualized as a weighted area-level aggregation of seven deprivation domain indices (DDIs): (1) income, (2) employment, (3) health and disability, (4) education, skills, and training, (5) barriers to housing and services, (6) crime, and (7) living environment. Most of the weight in the IMD comes from income and employment (45.00%) followed by health and education (27.00%) DDIs. The remaining DDIs contribute 9.30% weight each. Of note, the indicators used to construct DDIs in the IMD 2010 are different from those used in the 2011 census to classify households by deprivation dimension. **Barriers to housing and services** is composed of two sub-DDIs. First, “*wider barriers*” is a combined measure of numbers of homeless residents—as informed by the rate of acceptances for housing assistance under the homelessness provisions of the 1996 Housing Act—the proportion of households aged under 35 whose income means they are unable to afford to enter owner occupation, and the proportion of households judged to have insufficient space to meet the household’s needs (i.e., overcrowding). Second, “*geographical barriers sub-DDI*” is a combined measure of the mean distance to the closest general practitioner (GP) surgery, supermarket or general store, primary school, and post or sub-post office for people living in the LSOA. **Living environment DDI** is also composed of two sub-DDIs. First, *“indoors living environment*” measures the quality of housing as the combined proportion of homes that fail to meet the decent homes standard or do not have central heating. Second, “*outdoors living environment*” is a combined measure of air quality (based on modelled estimates of the atmospheric concentration of four pollutants) and road traffic accidents involving pedestrians and cyclists.

**The 2011 England and Wales Census [30]**

**Households by deprivation dimensions** is a less broad indicator of deprivation in England and Wales at the LSOA level. It classifies households by four deprivation dimensions, namely (1) employment (deprived if any member of the household [not a full-time student] is either unemployed or long-term sick), (2) education (deprived if no person in the household has at least level 2 education and no person aged 16–18 y is a full-time student), (3) health and disability (deprived if any person in the household has general health that is “bad or very bad” or has a long-term health problem), and (4) housing status (deprived if the household’s accommodation is either overcrowded with an occupancy rating −1 or less, is in a shared dwelling, or has no central heating). A household may be classified as being deprived in none or one to four of these dimensions in any combination. Study variables are based on the *“percentage of households deprived in one or several dimensions*” in each LSOA. **Population density** is the “number of persons per hectare” in each LSOA. **Health** variables are based on percentage of usual residents who, following self-assessment, reported “*bad/very bad general health*” or “*day-to-day activities ‘very’/’a little’ limited”* due to disability or a long-term health problem lasting more than 12 mo, in the census questionnaire. **Household overcrowding** variables are based on bedroom occupancy rating data in which the ages of the household members and their relationships to each other are used to derive the number of bedrooms they require. An “*occupancy rating of −1”* implies that a household has one fewer bedroom than required. An “*occupancy rating of −2 or less*” implies there are at least two fewer bedrooms than required. Variables for **usual residents living in communal establishments (CEs)** refer to the percentage of residents who live in managed residential accommodation with ten or more beds. “*Any communal establishment*” includes all CEs that meet the definition above. “*Communal care homes*” includes all medical and care CEs that are managed by local authorities or other and are classed as a “care home” with or without nursing. “*Other communal medical and care establishments”* include hospitals, mental health hospitals/units, children’s homes, and medical and care establishments other than care homes, which are managed by the National Health Service (NHS), local authorities, registered social landlord or housing associations, or other. **Ethnic group** variables relate to percentage of usual residents who reported “*White*,” “*Asian*,” “*Black*,” (including *Black-African* and *Black-Caribbean* subcategories) or “*Arab”* ethnicity in the census questionnaire. **Household spaces by dwelling type** are the percentage of households located in purpose-built blocks of flats or tenements as opposite to in, for example, detached or semidetached properties amongst other. **Length of residence in the UK** shows the percentage of usual residents who have lived in the UK “*less than 2 y*,” “*2 y or more but less than 5 y*,*”* or “*5 y or more but less than 10 y*.” It is derived from the date that a person last arrived to live in the UK and excludes short visits and usual residents born in the UK who have emigrated and since returned.

The Health and Social Care Information Centre (HSCIC, UK) provided hospital attendance data for catchment areas from the Hospital Episode Statistics (HES) Database [37]. This was defined as the percentage of usual residents of at least 1 y of age in each LSOA who had attended any London Accident and Emergency department or had been admitted to any London hospital (i.e., inside or outside catchment areas) as inpatients between 1 April 2011 and 31 March 2012.

### Statistical Analysis

Hierarchical models to investigate the spatial epidemiology of HA- and CA-MRSA were developed and fitted to the observed data using an integrated nested Laplace approximation (INLA) approach [38] in R-INLA package (www.r-inla.org) within R-3.1.1 statistical software [39]. INLA is a computationally efficient approach to Bayesian inference for latent Gaussian models. The latter are very wide and flexible class models that include spatial and spatial-temporal models [40,38]. Several examples in the literature have recently demonstrated the successful application of INLA to mapping of disease incidence and prevalence [41–43].

Based on counts of observed cases within LSOAs, we adopted a model-based approach to obtain reliable estimates of LSOA-level local risks of HA-, CA-, or any MRSA with or without consideration of available area-level statistics. A model-based approach provides a mechanism to “borrow information” across small LSOAs to improve local risk estimates, resulting in the smoothing of extreme risks based on small local sample sizes. It also enables consideration of the spatial setting by borrowing more information from neighbouring rather than distant areas and by smoothing local rates toward local (neighbouring) values where appropriate.

Numbers of MRSA cases in each LSOA were modelled as Poisson random variables by using a logarithmic link and accounting for the age-and-gender standardised expected count of MRSA cases in each LSOA. We considered models without covariates (i.e., disease mapping) and with covariates (i.e., ecological regression). To estimate local risks of MRSA, we examined models of increasing hierarchical complexity to account for the hierarchy of different populations in the analysis. We fitted models to the observed data with unstructured random effects only (independent random noise “iid” model), spatially structured random effects only (“Besag” model), and both structured and unstructured random effects (“Besag, York, and Mollie [BYM]” model) [44]. A model with unstructured random effects only induces some correlation among the observations but does not specifically induce spatial correlation. In other words, all the LSOA-specific risk estimates are a weighted average of the observed data in each LSOA and the global overall mean. On other hand, spatially structured random effects result in local estimates being a weighted average of the local data and an average of observations in neighbouring regions. Hence, such an approach borrows information locally, rather than globally. Structured random effects were modelled using an intrinsic conditional autoregressive structure (ICAR), in which neighbouring geographical areas for each LSOA were accounted for as described previously [45]. See S1 Methods for details. The set of neighbours for each LSOA was obtained from the shape file of the study region using the R packages “maptools” [46] and “spdep” [47,48].

We adopted uninformative priors for the model parameters of interest. We assigned vague normal priors for the fixed effects parameters with zero mean and large variance (equal to 10^4^) and exponential distributions with various rates for the structured and unstructured random effects precision parameters. For the “iid” and “Besag” models, priors on the precision corresponded to a gamma distribution with (1, 5e^-05^) shape and scale parameters respectively. For BYM, priors on the unstructured and structured effect precisions corresponded to a gamma distribution with parameters (1, 5e^-04^).

Structured random effects account for variation in the response variable that is due to the spatial arrangement of LSOAs, whilst unstructured effects account for unobserved heterogeneity that is not explained by the covariates or the spatial effects. For HA-, CA-, and any MRSA separately, the most appropriate random effect structure for disease mapping and ecological regression models was chosen based on examination of R-INLA built-in model selection tools in combination with inspection of the variation attributable to spatial effects and of the effects of different levels of spatial smoothing on the spatial field imposed by alternative prior parameters during prior sensitivity analyses. Model selection tools included the deviance information criterion (DIC) and the mean logarithmic score, and, given that DIC is accurate only when the number of effective parameters in the model is small compared to the total number of independent observations [49], the ratio of total observations (*n* = 513 LSOAs) to the number of effective parameters was also examined.

The potential for associations between MRSA and area-level socioeconomic and demographic profiles was examined in separate unadjusted ecological regression models and models adjusted by LSOA-level quintile-stratified hospital attendance data. Several transformations of each variable were examined, including untransformed (continuous) and two two-level dichotomous transformations reflecting classifications above and below the catchment area median or above and below a cut-off point that minimised the within group variance for each classification. Small-area-level estimates of MRSA and measures of association for the fixed terms (i.e., predictor variables), were summarized by relative risks (RRs). Significant predictor variables were those in which 95% credible intervals for MRSA RR estimates did not overlap with 1 in adjusted analyses. Out of all significant variables identified, we report the RR and 95% credible interval for the variable with lowest DIC within each group of variable transformations and/or “synonymous” variable indicators, to aid readability and interpretability of results. Synonymous variable indicators were considered for deprivation, health, household overcrowding, and usual residents by length of residency in the UK (S1 Table). For robustness, results from ecological regressions were validated across alternative random effect models and the range of prior scale parameters tested. Further details are given in S1 Methods.

Choropleth maps, in which areas are shaded in proportion to the quantile measurement of the variable being displayed, were used to show the posterior mean for LSOA-specific RRs of HA- and CA-MRSA compared to the whole catchment area and to show the distribution of significant covariates. Spatial correlations abound in disease mapping studies and can make the implementation of multiple regression impossible. In this study, we examined the effect of multiple indicators of social and material deprivation independently and following adjustment for hospital attendance data in each area. We then examined pairwise correlation coefficients between significant predictors of MRSA using the function “correlog” of the “ncf” package [50] to aid interpretability of adjusted and unadjusted results. A correlation network summarising the relationship between variables was produced using the “igraph” package [51].

### Ethics

This research was conducted following approval from the National Research Ethics Service (NRES Committee North West–Greater Manchester West; Research Ethics Committee [REC] reference 11/NW/0733). Approval and waived consent was obtained from NHS research and development departments at Guy’s and St Thomas’ NHS Foundation Trust, King’s College Hospital NHS Foundation Trust, and University Hospital Lewisham, now part of Lewisham and Greenwich NHS Trust.

## Results

### MRSA Cases in the Study Areas

Eight-hundred and thirty-nine MRSA positive patients were identified, of which 56.14% (*n* = 471) had a residential postcode within the three boroughs. Of the patients living in these boroughs, 79.41% (374/471) had MRSA identified in a single health care episode, whilst the remaining 20.59% (97/471) had MRSA identified in multiple episodes. Overall, 635 MRSA positive episodes in 471 individuals were identified, of which 43.31% (275/635) were inpatient, 39.37% (250/635) outpatient, and 17.32% (110/635) community episodes from GPs. MRSA cases were detected in 52.63% (270/513) of LSOAs in target boroughs.

Of the 471 individuals with MRSA with a residential postcode in the three boroughs, 83.23% (392/471) could be genotypically categorised into HA- or CA-MRSA based on genome sequencing, whilst the remaining 16.77% (79/471) were unclassified. 71.68% of classified cases (281/392) were HA-MRSA, and 26.28% (103/392) were CA-MRSA. Both CA- and HA-MRSA were identified in 2.04% of individuals (8/392). These proportions were similar to those observed for the whole study population (of 696/839 patients with MRSA genotypic characterisation, 74.28% had HA-MRSA (*n* = 517); 23.85% CA-MRSA (*n* = 166) and 1.87% mixed-MRSA (*n* = 13)).

### Population Structure in Catchment Areas

The number of usual residents in LSOAs ranged from 1,043 to 2,551, with substantial variation in age and gender population structure across areas (S2 Table). Only a minority of usual residents in the 513 LSOA in Lambeth, Southwark, and Lewisham were aged 65 y or more (between 0.58% and 18.77%), and 50.61% were female.

### Description of Area-Level Variables Considered in the Study

Summary statistics are given in S1 Table. There was substantial heterogeneity in the ethnicity makeup and factors of social and material deprivation across LSOAs, with ethnicities localising to different areas. The percentage of households deprived in 1–4 dimensions ranged from 26.64% to 83.33%, and the percentage of household spaces in purpose-built blocks of flats ranged from 1.10% to 97.70%. Cross-area differences were also evident for health indicators despite the relatively low percentage of residents aged 65 y or above in the same areas. The percentage of usual residents self-reporting bad or very bad health ranged from 0.48% to 10.13%, and that of residents reporting limited daily activities due to poor health ranged from 2.73% to 23.84%.

A large proportion of residents aged 1 y or older had attended a London hospital accident and emergency department or had been admitted to a hospital as an inpatient around the study period. The mean percentage of residents attending a hospital was 33.10% but ranged from 17.31% to 48.94% (S1 Table).

### Distribution of Genotypically Defined MRSA Types in Catchment Areas in Relation to Area-Level Hospital Attendance

For HA-MRSA (*n* = 281), and for all MRSA combined (HA-, CA- and unclassified; *n* = 471), the contribution of spatial (structured) effects to explaining the variance in RRs between LSOAs within the three boroughs was negligible (unadjusted model: 0.10%; model adjusted for area-level hospital attendance: 0.09%), with 99.90% variation attributable to unobserved heterogeneity alone (unstructured effects). In contrast, substantial variation in small area-level RRs of CA-MRSA within the three boroughs was attributable to the spatial arrangement of LSOAs (unadjusted model: 27.67%; adjusted model: 28.67%).

**Fig 2:**
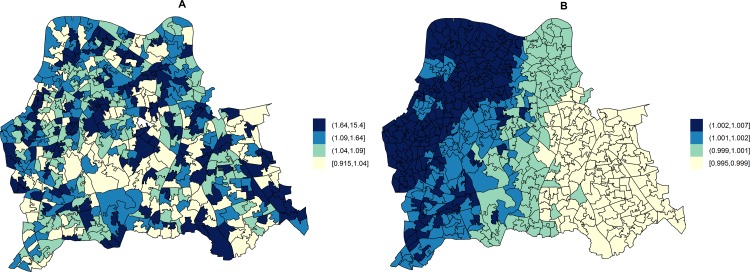
Maps for RR of HA- and CA-MRSA in LSOAs compared to the whole catchment area in disease mapping (unadjusted) models. Disease mapping models do not take into consideration the distribution and effect of risk factors for MRSA. These account for the observed and expected counts of HA- or CA-MRSA given the standardised age and gender population structure in each LSOA. HA-MRSA (A) was modelled considering unstructured random effects only (iid model). CA-MRSA (B) was modelled considering both unstructured and structured (spatial) random effects (BYM model). Cut-off values in figure legends correspond to quantiles for area-specific RRs of HA- and CA-MRSA, respectively.

In unadjusted models, the RR of HA-MRSA across the three boroughs ranged from 0.92 to 15.40 depending on the LSOA and that of CA-MRSA ranged from 1.00 to 1.01. Fig 2 shows the area-specific RRs of HA- and CA-MRSA compared to the whole catchment area in unadjusted models.

Area-level hospital attendance was an important predictor of the RR of MRSA. Specifically, the risk was only increased in areas where >37.16% of residents had attended hospital (fifth quintile), as compared to areas where hospital attendance was noted for ≤29.00% of usual residents (first quintile). In fifth quintile areas, the RR of HA-MRSA increased by 74.00% (1.74; 95% CI = 1.13–2.71) and that of CA-MRSA by 249.00% (2.49; 95% CI = 1.34–4.87).

Combining all MRSA (*n* = 471), the RR in catchment areas estimated from unadjusted models ranged from 0.84 to 8.39. The RR of any MRSA increased by 98.00% (1.98; 95% CI = 1.40–2.80) in areas where more than 37.16% of usual residents had attended a hospital (fifth quintile).

### Associations between Area-Level Social Determinants and Genotypically Defined MRSA Types

Ecological regression analyses to evaluate the effect of variables individually or after adjusting by quintile-stratified hospital attendance are shown in Tables 1 and 2 for HA-MRSA (*n* = 281) and CA-MRSA (*n* = 103), respectively. All significant predictors identified in adjusted analyses were also significant in unadjusted analyses. In addition, no variables were identified that were significant in adjusted analyses alone. HA- and CA-MRSA were both positively associated with household deprivation (i.e., percentage of households deprived in two to four dimensions or one to two dimensions), with black or black African ethnicities, and with a greater percentage of household spaces in purpose-built blocks of flats or tenements. In addition, HA-MRSA was positively associated with areas where greater percentages of usual residents had reported having very limited daily activities because of poor health and areas where greater percentages of usual residents resided in communal care homes.

**Table 1:** Ecological regression models for HA-MRSA in 513 LSOAs.

	Unadjusted					Adjusted						
	RR	95% CI	DIC	mD	pD	RR 5th Qi	95% CI	RR	95% CI	DIC	mD	pD
**Population Density**												
-	***-***	***-***	***-***	***-***	***-***	***-***	***-***	***-***	***-***	***-***	***-***	***-***
**Deprivation** ^¥^												
Households Deprived in 2–4 Dimensions (%)	1.53	1.15–2.03	980.59	874.06	106.52	1.15	0.66–2.03	1.57	1.06–2.33	981.68	872.53	109.15
**Health** ^¥^												
Usual Residents with Daily Activities Very Limited (%)	1.10	1.04–1.17	979.57	872.50	107.07	1.22	0.72–2.08	1.10	1.01–1.19	980.47	870.54	109.93
**Barriers to Housing**												
-	-	-	-	***-***	***-***	-	-	-	-	-	***-***	***-***
**Household Overcrowding** ^¥^												
-	-	-	-	***-***	***-***	-	-	-	-	-	***-***	***-***
**Environment**												
**- **	***-***	***-***	***-***	***-***	***-***	***-***	-	-	-	***-***	***-***	***-***
**Usual Residents Living in Communal Establishments (%)**												
Communal Care Homes (%)	1.24	1.12–1.37	975.90	874.08	101.82	1.61	1.05–2.50	1.24	1.12–1.37	975.14	872.06	103.08
**Usual Residents by Ethnic Group (%)**												
Any Black (%)	1.53	1.16–2.02	980.37	874.14	106.23	1.29	0.77–2.16	1.45	1.03–2.05	981.54	871.79	109.75
**Usual Residents by Length of Residency in the UK (%)** ^¥^												
**- **	-	-	-	***-***	***-***	-	-	-	-	-	***-***	***-***
**Household Spaces by Dwelling Type (%)**												
Household Spaces in Purpose-Built Block of Flats or Tenements (%)	1.01	1.00–1.01	980.81	873.77	107.04	1.52	0.97–2.38	1.01	1.00–1.01	980.62	871.19	109.43

A total of 281 HA-MRSA cases were identified in the study areas. Hierarchical Models with unstructured random effects (iid) were in all instances preferred over models with structured (spatial) random effects. The table shows variables that were found to be significant predictors of MRSA following adjustment for the quintile-stratified percentage of usual residents attending a hospital. All identified variables were also significant in unadjusted analyses. Only the lowest DIC model is presented in cases in which several indicator variables measuring the same overarching population trait were found to be significant. *RR 5* ^*th*^ *Qi* shows the RR of HA-MRSA in areas with more hospital attendance (fifth quintile) compared to in areas with less residents attending a hospital (first quintile). The RR for the covariate is shown in the “RR” column. Ninety-five percent credible intervals for RRs are also shown. The DIC is shown along with its two components summarising the fit (posterior mean of the deviance; *mD*) and complexity (number of effective parameters; *pD*) of the models.

^**¥**^ The variable indicator with lowest DIC is shown.

**Table 2:** Ecological regression models for CA-MRSA in 513 LSOAs.

	Unadjusted					Adjusted						
	RR	95% CI	DIC	mD	pD	RR 5th Qi	95% CI	RR	95% CI	DIC	mD	pD
**Population Density**												
-	***-***	***-***	***-***	***-***	***-***	***-***	***-***	***-***	***-***	***-***	-	***-***
**Deprivation** ^¥^												
Households Deprived in 1–2 Dimensions (%)	2.02	1.33–3.17	545.22	543.06	2.15	1.63	0.78–3.56	1.72	1.03–2.94	550.08	543.97	6.11
**Health** ^¥^												
-	-	-	-	***-***	***-***	-	-	-	-	-	-	***-***
**Barriers to Housing**												
Wider Barriers Sub-domain Score	1.98	1.33–2.99	544.97	542.82	2.15	2.00	1.05–3.98	1.76	1.16–2.70	547.12	541.02	6.10
**Household Overcrowding** ^¥^												
Households with bedroom occupancy rating of −1 (%)	1.84	1.24–2.73	546.94	544.79	2.15	1.97	1.02–3.98	1.58	1.04–2.41	549.75	543.65	6.10
**Environment**												
**-**	***-***	***-***	***-***	***-***	***-***	***-***	-	-	-	***-***	-	***-***
**Usual Residents Living in Communal Establishments (%)**												
-	-	-	-	***-***	***-***	-	-	-	-	-	-	***-***
**Usual Residents by Ethnic Group (%)**												
Black African (%)	2.10	1.40–3.19	543.06	540.91	2.15	1.53	0.73–3.32	1.86	1.15–3.10	547.99	541.89	6.10
**Usual Residents by Length of Residency in the UK (%)** ^¥^												
<2 y (%)	1.72	1.16–2.58	549.00	546.85	2.15	2.57	1.38–5.02	1.77	1.19–2.66	546.27	546.27	6.10
**Household Spaces by Dwelling Type (%)**												
Household Spaces in Purpose-Built Block of Flats or Tenements (%)	1.01	1.01–1.02	540.14	538.00	2.15	1.92	1.01–3.81	1.01	1.01–1.02	543.13	537.03	6.10

A total of 103 CA-MRSA cases were identified in the study areas. Hierarchical Models with unstructured plus structured (spatial) random effects (BYM) were in all instances preferred over models without spatial or unstructured random effects. The table shows variables that were found to be significant predictors of MRSA following adjustment for the quintile-stratified percentage of usual residents attending a hospital. All identified variables were also significant in unadjusted analyses. Only the lowest DIC model is presented in cases in which several indicator variables measuring the same overarching population trait were found to be significant. *RR 5* ^*th*^ *Qi* shows the RR of CA-MRSA in areas with more hospital attendance (fifth quintile) compared to in areas with less residents attending a hospital (first quintile). The RR for the covariate is shown in the “RR” column. Ninety-five percent credible intervals for RRs are also shown. The DIC is shown along with its two components summarising the fit (posterior mean of the deviance; *mD*) and complexity (number of effective parameters; *pD*) of the models.

^**¥**^ The variable indicator with lowest DIC is shown.

No indicators of poor health or residency in communal establishments (i.e., care homes) were positively associated with CA-MRSA. Instead, the RR of CA-MRSA was higher in areas with a greater percentage of overcrowded households and more deprivation according to the 2010 wider barriers score. The RR for CA-MRSA was also higher where more usual residents had immigrated to the UK within the 2 y preceding the 2011 census. The proportion of variance in CA-MRSA explained by the spatial structure component of the models presented in Table 2 ranged from 26.24% to 27.66%.

Analyses considering all 471 MRSA cases in catchment areas (Table 3) showed that variables significantly increasing the RR of any MRSA were a combination of HA-MRSA and CA-MRSA predictors (i.e., poor health, residency in care homes, and household overcrowding). A negative association was only identified in the combined analysis (*n* = 471). The RR of any MRSA decreased by about 30% in areas where more usual residents were of white ethnicity (36.30% unadjusted model; 26.20% adjusted model). See Table 3.

**Table 3:** Ecological regression models for all MRSA in 513 LSOAs.

	Unadjusted					Adjusted						
	RR	95% CI	DIC	mD	pD	RR 5^th^ Qi	95% CI	RR	95% CI	DIC	mD	pD
**Population Density**												
-	***-***	***-***	***-***	***-***	***-***	***-***	***-***	***-***	***-***	***-***	***-***	***-***
**Deprivation** ^¥^												
Households deprived in 2–4 dimensions (%)	1.64	1.32–2.04	1,286.15	1,170.96	115.19	1.39	0.91–2.13	1.49	1.12–1.99	1,286.70	1,168.97	117.73
**Health** ^¥^												
Usual Residents with Daily Activities Very Limited (%)	1.58	1.27–1.96	1,286.17	1,166.92	119.24	1.55	1.04–2.32	1.37	1.05–1.78	1,286.38	1,165.32	121.06
**Barriers to Housing**												
-	-	-	-	***-***	***-***	-	-	-	-	-	***-***	***-***
**Household Overcrowding** ^¥^												
Households with bedroom occupancy rating of −1 (%)	1.53	1.23–1.90	1,289.30	1,170.71	118.58	1.70	1.19–2.43	1.38	1.10–1.73	1,286.07	1,167.79	118.27
**Environment**												
**- **	***-***	***-***	***-***	***-***	***-***	***-***	-	-	-	***-***	***-***	***-***
**Usual Residents Living in Communal Establishments (%)**												
Communal Care Homes (%)	2.31	1.68–3.15	1,283.78	1,172.34	111.43	1.81	1.29–2.55	2.12	1.54–2.90	1,279.11	1,169.68	109.42
**Usual Residents by Ethnic Group (%)**												
Any Black (%)	1.65	1.34–2.05	1,285.05	1,169.71	115.34	1.45	0.97–2.17	1.47	1.13–1.91	1,285.50	1,167.43	118.08
Black African (%)	1.66	1.34–2.06	1,285.34	1,170.34	115.00	1.45	0.98–2.15	1.50	1.16–1.95	1,285.05	1,167.84	117.21
Any White (%)	0.64	0.51–0.79	1,286.74	1,167.58	119.16	1.55	1.04–2.32	0.74	0.57–0.96	1,286.49	1,165.44	121.05
**Usual Residents by Length of Residency in the UK (%)** ^¥^												
**- **	-	-	-	***-***	***-***	-	-	-	-	-	***-***	***-***
**Household Spaces by Dwelling Type (%)**												
Household Spaces in Purpose-Built Block of Flats or Tenements (%)	1.01	1.00–1.01	1,286.96	1,168.83	118.14	1.71	1.21–2.44	1.01	1.00–1.01	1,283.39	1,166.27	117.11

A total of 471 MRSA cases were identified in the study areas. Hierarchical Models with unstructured random effects (iid) were in all instances preferred over models with structured (spatial) random effects. The table shows variables that were found to be significant predictors of MRSA following adjustment for the quintile-stratified percentage of usual residents attending a hospital. All identified variables were also significant in unadjusted analyses. Only the lowest DIC model is presented in cases in which several indicator variables measuring the same overarching population trait were found to be significant. *RR 5* ^*th*^ *Qi* shows the RR of any MRSA in areas with more hospital attendance (fifth quintile) compared to in areas with less residents attending a hospital (first quintile). The RR for the covariate is shown in the “RR” column. Ninety-five percent credible intervals for RRs are also shown. The DIC is shown along with its two components summarising the fit (posterior mean of the deviance; *mD*) and complexity (number of effective parameters; *pD*) of the models.

^**¥**^ The variable indicator with lowest DIC is shown.

**Fig 3:**
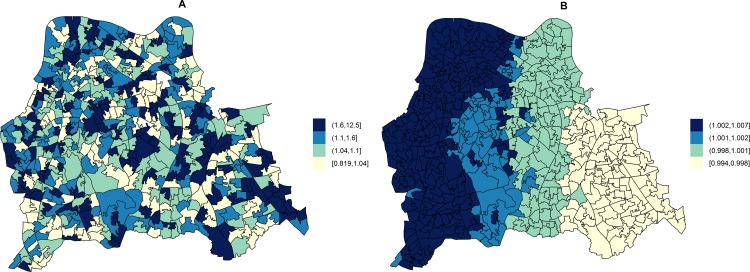
Maps for RR of HA- and CA-MRSA in LSOAs compared to the whole catchment area, in ecological regression models accounting for area-specific quintile-stratified percentage of usual residents attending a hospital and households deprived in 2–4 dimensions (HA-MRSA) or 1–2 dimensions (CA- MRSA). Ecological regression models account for the distribution and effect of risk factors for MRSA in addition to the observed and expected counts of HA- or CA-MRSA given the standardised age and gender population structure in each LSOA. HA-MRSA (A) was modelled considering unstructured random effects only (iid model). CA-MRSA (B) was modelled considering both unstructured and structured (spatial) random effects (BYM model). Cut-off values in figure legends correspond to quantiles for area-specific RRs of HA- and CA-MRSA, respectively.

The area-specific RRs of MRSA compared to the whole catchment area in adjusted models accounting for hospital attendance and households deprived in 2–4 dimensions (HA-MRSA) or 1–2 dimensions (CA-MRSA) are shown in Fig 3. The overall RR of HA-MRSA ranged from 0.82 to 12.46 depending on the LSOA and that of CA-MRSA from 0.99 to 1.01. The RR for all MRSA combined (*n* = 471) in a model adjusted for hospital attendance and households deprived in 2–4 dimensions ranged from 0.76 to 6.70 depending on the LSOA.

### Correlations of Area-Level Predictors of Genotypically Defined MRSA Types

**Fig 4:**
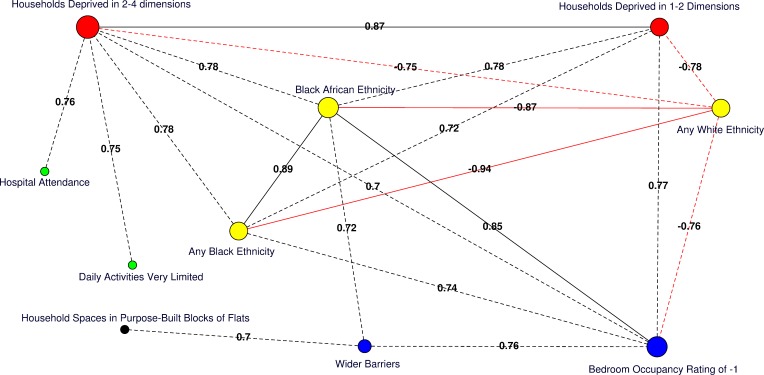
Correlation network of area-level significant predictors of MRSA infection or colonisation in catchment areas of the London hospital cohort. Moderate positive PCCs are shown by black dashed edges (≥0.70 and ≤0.80) and strong PCCs by continuous black edges (>0.80). Moderate negative PCCs are shown by red dashed edges (≥−0.80 and ≤−0.70) and strong negative PCCs by continuous red edges (<−0.80). The size of vertices reflects the number of variables correlated with that vertex. Vertex colours represent variable indicators of health (green), deprivation (red), ethnicity (yellow), wider barriers and household overcrowding (blue), and household spaces by dwelling type (black). Areas with more households deprived in any dimension are those where more persons of black or black African ethnicity reside and those with greater percentages of overcrowded households. The later also correspond to those where more household spaces are located in purpose-built blocks of flats or tenements and to those with worse wider barriers scores. Negative PCCs show that persons of white ethnicity tend to reside outside the boundaries of the deprived areas where usual residents of black or black African ethnicity predominate. More residents from deprived areas self-report having daily activities very limited because of poor health, have attended a London accident and emergency hospital department, or have been admitted to hospital as inpatients around the time of the study. The greatest number of moderate to strong correlations are for the percentage of households deprived in 2–4 dimensions (*n* = 7), bedroom occupancy rating of –1 (*n* = 6), and black African ethnicity (*n* = 6). Maps for predictor variables of HA-, CA-, and any MRSA are shown in Fig 5.

Moderate (Pearson correlation coefficient [PCC] ≥ 0.70 or ≤ −0.70) to strong (PCC > 0.80 or < −0.80) correlations amongst significant predictors of HA-MRSA, CA-MRSA, or any MRSA are presented for the catchment areas in Fig 4. Of note, the percentage of usual residents living in communal care homes or having moved to the UK within the 2 y preceding the 2011 census were not correlated with deprivation or any other significant predictors of MRSA.

**Fig 5:**
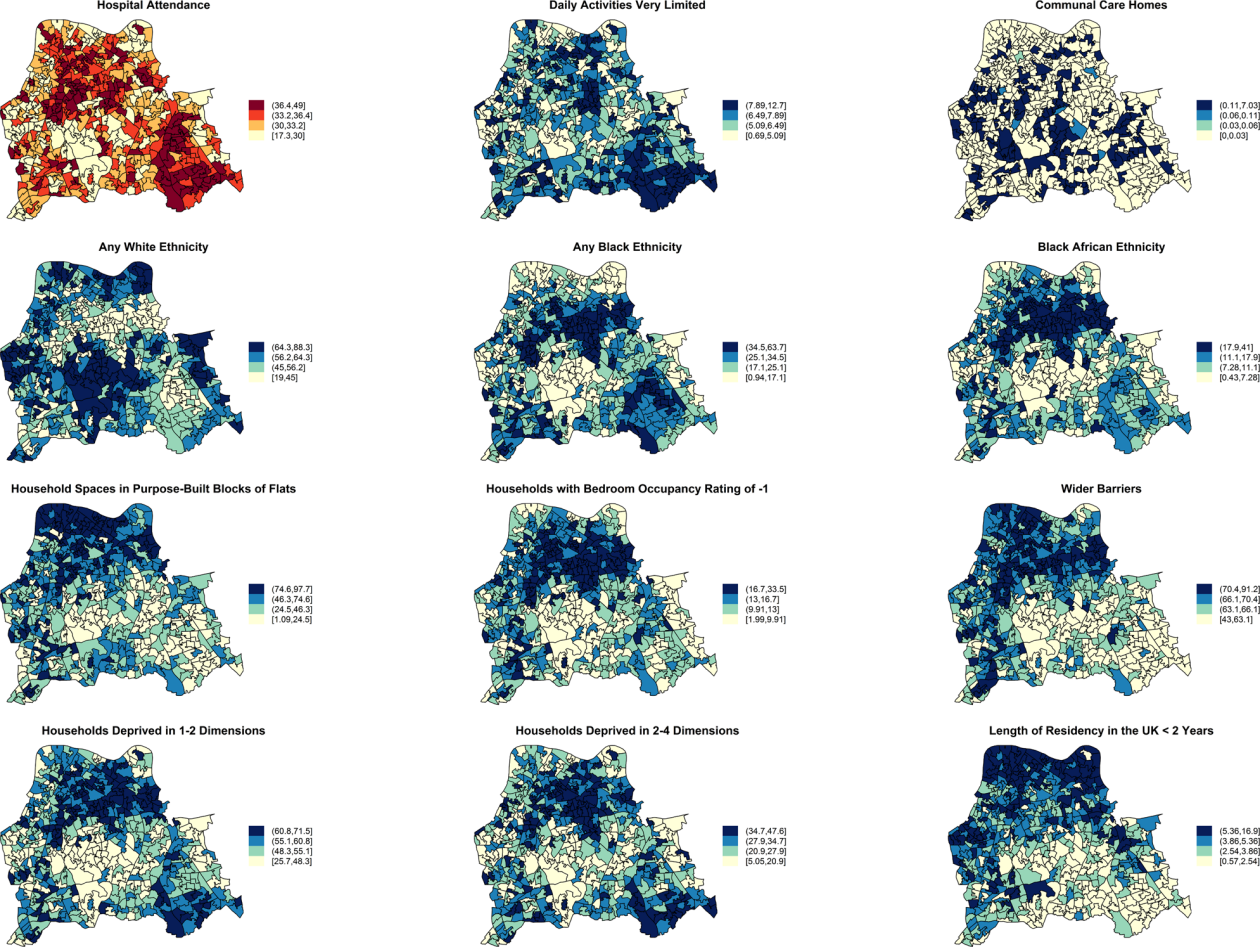
Maps for significant predictors of MRSA infection or colonisation in catchment areas of the London hospital cohort. The brown coloured map shows the percentage of usual residents of at least 1 y of age in each LSOA who attended any London accident or emergency department or had been admitted to any London hospital between 1 April 2011 and 31 March 2012. Blue coloured maps show the spatial distribution of population traits associated with either lower or increased risk of MRSA infection or colonisation following adjustment for hospital attendance data. The cut-off values in figure legends correspond to the variable quantiles. The units in the legends correspond to percentage of usual residents (with daily activities very limited; living in communal care homes; of any white, black, or black African ethnicity; with length of residency in the UK <2 y), percentage of households or household spaces (in purpose-built blocks of flats; with bedroom occupancy rating of −1; deprived in 2–4 or 1–2 dimensions) and a score, which is a combined measure of household overcrowding, homelessness, and difficulty of access to owner occupation (wider barriers).

## Discussion

To the best of our knowledge, this is the first study to use spatial models to describe the epidemiology of MRSA and to investigate genetically defined CA- and HA-MRSA classifications separately in relation to social determinants and transmission niches. Multiple reports had previously linked MRSA rates to postcode or neighbourhood data [52–59], but only classical tests [4,54], nonspatial regression [52,53,55–61], or cluster detection methods [52,58] had been used to determine the impact of socioeconomic indicators. Here for the first time, we provide unprecedented insight into the epidemiology of HA- and CA-MRSA in the UK, by accounting for spatial effects—and hence the potential for impact from neighbouring geographies—and confounding hospital attendance.

We specifically conducted the study in three adjacent boroughs of South East London to take advantage of the wide variation that exists in population traits across LSOAs. Our work suggests that the most deprived areas are at greater risk of MRSA but that the causality of this association differs depending on the MRSA genetic classification. We hypothesise that relevant risk factors must lie within the deprivation domains considered in the 2011 census (i.e., households deprived in any dimension). In contrast, a lack of association with the 2010 IMD overall score suggests that such a wide scoring predominantly weighted by income and employment (45.00%) with lesser contribution of wider barriers and household overcrowding is less helpful at describing the spatial epidemiology of MRSA in the UK.

Vast analytical complexity arises from disease mapping studies in which spatial correlations abound, which can result in spurious associations. We found that areas with a higher percentage of deprived households correlated closely with various other identified risk factors for MRSA, especially black or black African ethnicity, poor housing, poor health, and hospital attendance. In this context, we sought to address whether the most important precursor for acquiring MRSA is living in deprived conditions or attending a hospital as a result of being deprived and whether it is the hospital or the community that presently constitutes the main transmission site for MRSA.

We found that 26.24% to 28.67% of area-level variation in CA-MRSA could be explained by the spatial arrangement of LSOAs, implying that residents in locations neighbouring high-risk areas are themselves at higher risk of infection and/or colonisation compared to more distant locations. In contrast, no spatial patterns were observed for HA-MRSA, suggesting that little or no transmission of hospital lineages occurs in the community. These findings indicate that the predominant transmission route for genetically classified CA-MRSA in the UK is still the community, whilst transmission of HA-MRSA continues to be hospital led, with little evidence for transmission outside the health care or care home setting. The implications are 2-fold. The first implication is that an association between HA-MRSA and deprivation may result from the increased likelihood of attending a hospital as a result of being deprived rather than from living in deprived conditions alone. This is supported by additional markers of poor health (i.e., daily activities very limited) and the percentage of usual residents living in communal care homes being significant predictors for HA-MRSA and by the lack of an independent association with wider barriers and/or household overcrowding alone. Of note, previous studies reporting a link between socioeconomic deprivation and *S*. *aureus* bacteraemia [56], postoperative MRSA infection [4], or HA-MRSA [55] had not adjusted for area-level attendance at hospitals. Secondly, there is an implication that an association between CA-MRSA and deprivation most likely arises from living in deprived conditions, rather than from attending a hospital as a result of being deprived. This is supported by the lack of association between CA-MRSA and markers of poor health (e.g., daily activities very limited because of poor health) or residency in care homes. Instead, CA-MRSA is associated with wider barriers (a combination of homelessness, low income, and household overcrowding) and with the percentage of households with bedroom occupancy rates reflecting overcrowding. We hypothesise that in the UK an association with hospital attendance may be spurious, due to more persons in deprived areas attending a hospital, although evidence of ongoing or limited transmission of CA-MRSA in the hospital setting is lacking. CA-MRSA has previously been linked to socioeconomic deprivation [54,55,57,58,60], public housing [52], and alternative housing (e.g., shelters) [53,59] in studies outside the UK. A link between CA-MRSA and household overcrowding has been shown in some non-UK studies [54,61] but not others [52].

These findings are important because whilst the overall burden of MRSA in Europe is declining [62], due to decline in HA-MRSA lineages [23,63,64], CA-MRSA lineages are emerging in every continent [17,65,66]. The prevalence of CA-MRSA in Europe is thought to be increasing [7] partly through continuous importation from endemic regions [67,68]. Our work suggests that the prevalence of CA-MRSA in South East London is significant (26.28% of cases were CA-MRSA), and may be increasing based on observations that community-level transmission from deprived areas to neighbouring areas is ongoing and that importation of CA-MRSA is linked with recent immigration to the UK.

An advantage to our study is that it has provided a representative sample of usual residents receiving care in the catchment areas. The vast majority of biological samples from public and private patients residing in the study areas are analysed by laboratories in the cohort, whilst the contribution of other diagnostic providers is negligible. This is in contrast to studies in which a sample may be restricted to a particular population layer, such as patients with medical insurance. There are several limitations to our study. Firstly, analyses of aggregated data risk the “ecological fallacy” whereby relationships apparent at the group level are spuriously assumed to operate at the individual level. This bias is a result of the fact that, unlike individual‐level studies, group‐level studies do not link individual outcome events to individual exposure histories [69,70]. We therefore recognise the need for parallel analysis of individual level data to revisit the impact of socioeconomic deprivation and demographic indicators on risk of HA- and CA-MRSA infection or colonisation in Europe. Secondly, choosing small values for the scale parameter of priors, the LSOA-specific RRs for CA-MRSA compared to the whole catchment area, suggested an almost constant risk across the area (RRs: 0.99–1.01). The apparent “flat risk,” however, almost undoubtedly resulted from the very low numbers of observed CA-MRSA cases in each LSOA (minimum = 0; maximum = 3) and from comparing individual LSOA RRs against the average as opposite to against other/neighbouring LSOAs. Increasing the scale parameter for the prior on the spatial effect precision, and hence increasing the weight of small observations, significant differences in CA-MRSA LSOA-specific RRs were apparent, with LSOA-specific posterior probabilities indicating excess risk for some of the areas. Moreover, greater percentage variation attributable to spatial effects was also observed at greater values of the scale parameter. This was in sharp contrast with HA-MRSA, with which effects of different levels of spatial smoothing on the spatial field imposed by alternative prior parameters had little influence on LSOA RRs, and percentage variation attributable to spatial effects remained negligible regardless of prior choice. Consequently, we argue that, unlike HA-MRSA, the distribution of CA-MRSA cases was spatially structured.

In summary, this study shows that the predominant transmission niches for HA- and CA-MRSA in South East London are specific to each genetic classification. HA-MRSA lineages originate from hospitals, and there is no evidence that active transmission of these lineages occurs outside the health care setting. We present evidence that CA-MRSA strains are spreading in the community from the most deprived areas where favourable conditions are met. There is also importation from recent immigration. The extent to which CA-MRSA is transmitting within health care premises is unknown, particularly in the context of declining overall MRSA prevalence in UK hospitals due to stringent control measures. However, in some countries, CA-MRSA is increasingly implicated in nosocomial infections [13,16], has begun to spread within hospitals [13], and may have the capacity to displace HA-MRSA in these settings [71–73], particularly given repeated community admissions [26] coupled with poor adaptability of HA-MRSA to persist in the community [21,63]. In this context, we show that factors are at play in the UK that could result in the displacement of HA-MRSA lineages in favour of CA-MRSA in the future. We propose that future efforts to master sustained control of MRSA in hospitals and the community should focus on prevention of community spread within deprived areas. Future reviews of UK hospital admission screening policies for MRSA should carefully consider the growing threat of importation of CA-MRSA lineages into hospitals.

## Supporting Information

S1 Methods Description of modelling approach. (DOCX)

S1 Table Summary statistics for area-level variables in 513 LSOAs within catchment areas of the hospital cohort. Data was obtained from the 2011 England and Wales census [30] unless otherwise specified. ^**1**^The English Indices of Deprivation 2010 data [36]. ^**2**^HSCIC data.
(DOC)

S2 Table Population structure of 513 LSOAs within catchment areas of the hospital cohort. Data from the 2011 England and Wales census [30].
(DOC)

S1 Text Individual patient-level metadata. (XLS)

S2 Text LSOA-level aggregated metadata. (XLS)

## Abbreviations

BYM: Besag, York, and Mollie’s model
CA: community associated
CE: communal establishment
CIDR: Centre for Clinical Infection and Diagnostics Research
DDI: Deprivation Domain Index
DIC: deviance information criterion
GP: general practice or practitioner
GSTT: Guy’s and St Thomas’ NHS Foundation Trust
HA: health care associated
HES: Hospital Episode Statistics
HSCIC: Health and Social Care Information Centre
ICAR: intrinsic conditional autoregressive structure; iid, independent random noise model
IMD: Index of Multiple Deprivation
INLA: integrated nested Laplace approximation
LSOA: Lower Layer Super Output Area
MLST: multilocus sequence type
MRSA: methicillin-resistant *Staphylococcus aureus*
NHS: National Health Service
NIHR: National Institute for Health Research
NRES: National Research Ethics Service
PCC: Pearson correlation coefficient
PVL: Panton-Valentine leucocidin
REC: Research Ethics Committee
RR: relative risk
SCCmec: staphylococcal cassette chromosome *mec*
UK: United Kingdom
WGS: whole genome sequencing
