# Supplementary material for: Evidence for Community Transmission of Community-Associated but Not Health-Care-Associated Methicillin-Resistant Staphylococcus Aureus Strains Linked to Social and Material Deprivation: Spatial Analysis of Cross-sectional Data
Source: PLoS Med. 2016 Jan 26;13(1):e1001944. doi: 10.1371/journal.pmed.1001944 (PMC4727805; doi:10.1371/journal.pmed.1001944)
Supplement: S1 Methods — (DOCX) [file pmed.1001944.s001.docx]

**S1 METHODS**

We now give details of our modelling approach. We largely follow the terminology and setting of Wakefield, Best and Waller (2000) [1]. Let $Y_{ij}$ and $N_{ij}$represent the number of cases and the number of individuals at risk in stratum$j$, $j=1,...,J$and LSOA $i$, $i=1,...N$. It is important to stratify the data by known risk factors (e.g. age and sex) since different LSOAs may contain different proportions of individuals within each stratum and ignoring this information may lead to spurious conclusions. A starting point of an analysis for such kind of data is to assume a Binomial model, where $Y_{ij}\sim Bin(N_{ij},p_{ij})$ where $p_{ij}$ denotes the risk of MRSA in area $i$ and stratum$j$. However, under the ``rare disease assumption'' which requires $N_{ij}$ to be large and $p_{ij}$ small, the Binomial model can be approximated by a Poisson model where $Y_{ij}\sim Po(N_{ij}p_{ij})$. Although in principle one can obtain maximum likelihood estimates for the area-specific risks by $Y_{ij}/N_{ij}$ in practice, the data will be too sparse to obtain precise estimates for each of these $NxJ$ parameters. Therefore some simplifying assumptions are required.

One of the most commonly made assumptions is the one in which$p_{ij}=\vartheta_{i}p_{j}$, also known as the *proportionality assumption*. Under this assumption, $\vartheta_{i}$ corresponds to the relative risk of disease in LSOA $i$ with respect to the reference rate in each stratum$j$. The great advantage of the Poisson approximation is that combined with the proportionality assumption we can sum over strata and obtain$Y_{i}\sim Po(E_{i}\vartheta_{i})$, where $Y_{i}=\sum_{j} Y_{ij}$ and $E_{i}=\sum_{j} N_{ij}p_{j}$ denotes the expected number of cases in area $i$ with strata-specific references rate $p_{j}$.

The above procedure is known as standardisation and in this particular study we have done the standardisation by age and gender. Under this model formulation one can estimate the standardised incidence ratio (SIR) in a straightforward manner by$Y_{i}/E_{i}$. However, the SIR has several limitations. For example, the variance of this estimator is inversely proportional to the population size of the $i_{th}$ LSOA which means that the more extreme relative risks are usually found in LSOAs with small populations. A further disadvantage is that information from other LSOAs is completely ignored.

One way to overcome the limitations of SIR is to build a model for the relative risk for the $i_{th}$ LSOA as a function of LSOA-specific risk factors, say$X_{i}$. We specify the following log-linear model as follows$\log(\vartheta_{i})=a+bX_{i},i=1,...,N$. Even when accounting for LSOA-specific risk factors, the estimates of the relative risks, $\vartheta_{i}$, will be highly unstable due to the amount of data available in small LSOAs. One can aid the estimation by specifying a joint model for $\vartheta=(\vartheta_{1},\vartheta_{2},...,\vartheta_{N})$ which will allow the estimate of each $\vartheta_{i}$ to “borrow strength” from the rest estimates, $(\vartheta_{1},\vartheta_{2},...,\vartheta_{i-1},\vartheta_{i+1},...,\vartheta_{N})$. This can be achieved by specifying a multivariate probability distribution for $\vartheta$. There are many different ways to do this and they usually differ on the form of variability in the $\vartheta_{i}$.

In this work we considered models of increasing complexity. In particular, having accounted for an LSOA-specific risk factor $X_{i}$, we fitted hierarchical models to the observed with unstructured random effects only (“iid model”), spatially structured random effects only (“Besag model”) and both unstructured and spatially structured random effects (“BYM model”). The “BYM model” decomposes the relative risk as follows:

$$\log\left( \vartheta_{i} \right)= \alpha+ \beta X_{i}+u_{i}+v_{i}, i=1, \ldots, N$$

where

$u_{i} \sim Normal(0, {1/\tau}_{u}$)

$v_{i}|v_{-i} \sim Normal(\sum_{j\sim i} \frac{v_{j}}{n_{i}}, {1/(n}_{i}\tau_{v})$)

where $n_{i}$ denotes the number of neighbouring areas of LSOA $i$ and $j\sim i$ means that LSOA $j$ is adjacent to LSOA $i$. The precisions for the unstructured and structured random effects are denoted by $\tau_{u}$ and $\tau_{v}$ respectively.

To examine the relationship between socio-economic deprivation and MRSA, we set to identify population traits that explain cross-area extra variation in HA- and CA-MRSA relative risks (RRs), following adjustment for hospital attendance data. We addressed our objectives by adhering to a nine-step analysis plan for HA-MRSA, CA-MRSA and any MRSA separately.

**First**, we fitted a model without covariates (herein referred to as ‘disease mapping model’). We decided on the most appropriate random effect structure (i.e. “iid”, “Besag” or “BYM”) based on several model evaluation statistics (including deviance information criterion [DIC], mean logarithmic score, ratio of the total number of independent observations to the number of effective parameters) and the percentage variation attributable to the spatial effects.

**Second**, we conducted prior sensitivity analysis for disease mapping models by modifying the scale parameter for Gamma priors on the structured (where appropriate) and unstructured effect precisions as: (1, 1e-03) and (1, 1e-02).

**Third**, we prepared the variables that were to be considered in ecological regression analyses. For each variable listed in Supplementary Table 1 (n=33 variables), we created three variables:

- - 1. A continuous (untransformed) variable (“C”).
    2. A dichotomous transformation reflecting classification of variable values above or below the catchment area median (“M”).
    3. A dichotomous transformation classifying variable values above or below a cut-off point which minimises the within group variance for each classification (“V”).

**Fourth**, for each variable (n=99), we fitted ecological regression models adjusted by quintile-stratified hospital attendance data with the preferred random effect structure as informed by steps One and Two. We then retrieved the list of significant predictors – defined as those were 95% CI for estimated RRs did not overlap with one - along with model evaluation statistics and the percentage variation attributable to spatial effects where appropriate.

**Fifth**, we repeated the analyses as described in step Four, but fitted second best a random effect structures.

**Sixth,** for each of the ecological regression analyses above, we conducted prior sensitivity analysis for preferred and alternative random effect structures.

**Seventh**, by comparing model evaluation statistics and the percentage variation attributable to spatial effects, we examined whether the preferred random effect structure (see step One) was still the most reasonable / appropriate for significant regressions identified in steps Four to Six.

**Eighth**, out of all significant variables identified, we report the relative risk (RR) and 95% confidence interval for the variable with lowest DIC within each group of variable transformations and/or ‘synonymous’ variable indicators, to aid readability and interpretability of results. Synonymous variable indicators were considered for deprivation, health, household overcrowding and usual residents by length of residency in the UK (S1 Table).

**Ninth**, we confirmed that all adjusted significant predictors presented in the manuscript (see step Eight) were also significant in unadjusted analyses. We did not identify any variables that were significant following adjustment for hospital attendance data but not in univariate analyses.

The preferred random effect structure for each MRSA classification was supported for the range of prior parameters tested. Similarly, the conclusions from ecological regression analyses were consistent across all random effect models and prior parameters tested.

**Bibliography**

1. Wakefield J, Best N, Waller L. Bayesian approaches to disease mapping. In: Elliott, Wakefield, Best, Briggs, editors. Spatial Epidemiology: Methods and Applications. Oxford University Press; 2000. pp. 104–127. Available: http://www.oup.co.uk/isbn/0-19-851532-4
