# Supplementary material for: Evidence for Community Transmission of Community-Associated but Not Health-Care-Associated Methicillin-Resistant Staphylococcus Aureus Strains Linked to Social and Material Deprivation: Spatial Analysis of Cross-sectional Data
Source: PLoS Med. 2016 Jan 26;13(1):e1001944. doi: 10.1371/journal.pmed.1001944 (PMC4727805; doi:10.1371/journal.pmed.1001944)
Supplement: S1 Table — Data was obtained from the 2011 England and Wales census [30] unless otherwise specified. 1The English Indices of Deprivation 2010 data [36]. 2HSCIC data. (DOC) [file pmed.1001944.s002.doc]

|  | **Min** | **1^st^ Qu.** | **Median** | **Mean** | **3^rd^ Qu.** | **Max** |
| --- | --- | --- | --- | --- | --- | --- |
| ***Deprivation*** |  |  |  |  |  |  |
| Index of multiple deprivation score ^1^ | 7.36 | 24.12 | 31.33 | 30.60 | 37.34 | 54.21 |
| Households deprived in 1-4 dimensions (%) | 26.64 | 53.70 | 63.50 | 61.98 | 71.38 | 83.33 |
| Households deprived in 2-4 dimensions (%) | 5.05 | 20.85 | 27.87 | 27.65 | 34.74 | 47.65 |
| Households deprived in 1-2 dimensions (%) | 25.72 | 48.29 | 55.08 | 53.95 | 60.79 | 71.53 |
| Households deprived in 2-3 dimensions (%) | 5.05 | 20.10 | 26.75 | 26.64 | 33.44 | 45.14 |
| Households deprived in 3-4 dimensions (%) | 0.45 | 5.53 | 7.87 | 8.03 | 10.64 | 16.07 |
| ***Barriers to Housing*** |  |  |  |  |  |  |
| Barriers to housing and services domain score ^1^ | 25.51 | 33.79 | 35.61 | 36.08 | 38.16 | 53.58 |
| Geographical barriers sub-domain score ^1^ | 0.03 | 1.33 | 3.36 | 5.25 | 6.80 | 39.78 |
| Wider barriers sub-domain score ^1^ | 43.03 | 63.08 | 66.10 | 66.91 | 70.39 | 91.22 |
| ***Environment*** |  |  |  |  |  |  |
| Living environment deprivation domain score ^1^ | 10.17 | 34.71 | 42.46 | 42.63 | 50.79 | 79.03 |
| Indoors living environment sub-domain score ^1^ | 2.05 | 25.96 | 33.08 | 34.71 | 43.4 | 80.85 |
| Outdoors living environment sub-domain score ^1^ | 11.81 | 40.26 | 58.46 | 58.47 | 75.64 | 100.00 |
| ***Population Density*** |  |  |  |  |  |  |
| Number of Persons per hectare | 14.40 | 85.40 | 109.40 | 119.95 | 146.20 | 344.60 |
| ***Health*** |  |  |  |  |  |  |
| Usual residents with daily activities very limited (%) | 0.70 | 5.10 | 6.50 | 6.63 | 7.90 | 12.7 |
| Usual residents with daily activities very or ‘a little’ limited (%) | 2.73 | 11.34 | 13.48 | 13.61 | 15.85 | 23.84 |
| Usual residents with very bad health (%) | 0.00 | 0.90 | 1.20 | 1.24 | 1.60 | 3.10 |
| Usual residents with bad or very bad health (%) | 0.48 | 3.94 | 4.84 | 4.96 | 6.02 | 10.13 |
| ***Hospital Attendance*** |  |  |  |  |  |  |
| Usual residents (> 1 year of age) admitted to hospital or who attended AE (%) ^2^ | 17.31 | 30.03 | 33.21 | 33.10 | 36.43 | 48.94 |
| ***Household Overcrowding*** |  |  |  |  |  |  |
| Households with bedroom occupancy rating of -1 (%) | 2.00 | 9.90 | 13.00 | 13.56 | 16.70 | 33.50 |
| Households with bedroom occupancy rating of ≤ -2 (%) | 0.13 | 1.29 | 2.01 | 2.35 | 3.10 | 10.00 |
| ***Usual Residents Living in Communal Establishments*** |  |  |  |  |  |  |
| Any communal establishment (%) | 0.00 | 0.00 | 0.00 | 1.30 | 0.80 | 35.60 |
| Communal care homes (%) | 0.00 | 0.00 | 0.00 | 0.38 | 0.12 | 7.03 |
| Other communal medical and care establishments (%) | 0.00 | 0.00 | 0.00 | 0.07 | 0.00 | 3.91 |
| ***Usual residents by Ethnic Group*** |  |  |  |  |  |  |
| Any White (%) | 19.03 | 44.98 | 56.18 | 55.08 | 64.36 | 88.35 |
| Any Asian (%) | 2.01 | 5.64 | 7.45 | 8.55 | 10.16 | 35.39 |
| Any Black (%) | 0.95 | 17.12 | 25.08 | 26.56 | 34.54 | 63.65 |
| Black: African (%) | 0.43 | 7.29 | 11.09 | 13.15 | 17.87 | 40.96 |
| Black: Caribbean (%) | 0.16 | 5.59 | 8.95 | 8.98 | 11.88 | 23.59 |
| Other: Arab (%) | 0.00 | 0.32 | 0.53 | 0.64 | 0.84 | 3.48 |
| ***Usual Residents by Length of Residency in the UK*** |  |  |  |  |  |  |
| < 2 years (%) | 0.57 | 2.55 | 3.87 | 4.34 | 5.37 | 16.92 |
| ≥ 2 and < 5 years (%) | 2.71 | 6.20 | 9.25 | 9.78 | 11.91 | 29.96 |
| ≥ 5 and < 10 years (%) | 6.11 | 13.38 | 17.53 | 17.93 | 21.29 | 44.15 |
| ***Household Spaces by Dwelling Type*** |  |  |  |  |  |  |
| Household spaces in purpose-built block of flats or tenements (%) | 1.10 | 24.50 | 46.30 | 48.66 | 74.60 | 97.70 |
