# Supplementary material for: Evidence for Community Transmission of Community-Associated but Not Health-Care-Associated Methicillin-Resistant Staphylococcus Aureus Strains Linked to Social and Material Deprivation: Spatial Analysis of Cross-sectional Data
Source: PLoS Med. 2016 Jan 26;13(1):e1001944. doi: 10.1371/journal.pmed.1001944 (PMC4727805; doi:10.1371/journal.pmed.1001944)
Supplement: S2 Table — Data from the 2011 England and Wales census [30]. (DOC) [file pmed.1001944.s003.doc]

| **Age and Gender Population Structure** | **Min** | **1^st^ Qu.** | **Median** | **Mean** | **3^rd^ Qu.** | **Max** |
| --- | --- | --- | --- | --- | --- | --- |
|  |  |  |  |  |  |  |
| **Usual residents (n)** | 1043 | 1521 | 1658 | 1691 | 1838 | 2551 |
| 0 – 14 years old (%) | 4.41 | 15.02 | 18.17 | 18.02 | 21.21 | 31.99 |
| 15 – 64 years old (%) | 60.36 | 68.93 | 72.95 | 73.63 | 77.30 | 94.07 |
| ≥ 65 years old (%) | 0.58 | 6.22 | 8.02 | 8.35 | 10.06 | 18.77 |
| **Male residents (%)** | 42.53 | 47.66 | 49.17 | 49.39 | 50.70 | 60.18 |
| 0 – 14 years old males (%) | 3.63 | 14.94 | 18.77 | 18.75 | 22.57 | 37.70 |
| 15 – 64 years old males (%) | 57.14 | 68.86 | 73.71 | 73.86 | 78.09 | 94.92 |
| ≥ 65 years old males (%) | 0.28 | 5.59 | 7.16 | 7.38 | 8.97 | 17.37 |
| **Female residents (%)** | 39.82 | 49.30 | 50.83 | 50.61 | 52.34 | 57.47 |
| 0 – 14 years old females (%) | 4.45 | 14.80 | 17.71 | 17.36 | 20.11 | 31.55 |
| 15 – 64 years old females (%) | 60.47 | 69.09 | 72.62 | 73.35 | 76.69 | 93.57 |
| ≥ 65 years old females (%) | 1.00 | 6.90 | 8.89 | 9.29 | 11.40 | 21.22 |
|  |  |  |  |  |  |  |
